# Mice lacking caspase-2 are protected from behavioral changes, but not pathology, in the YAC128 model of Huntington disease

**DOI:** 10.1186/1750-1326-6-59

**Published:** 2011-08-19

**Authors:** Jeffrey B Carroll, Amber L Southwell, Rona K Graham, Jason P Lerch, Dagmar E Ehrnhoefer, Li-Ping Cao, Wei-Ning Zhang, Yu Deng, Nagat Bissada, R Mark Henkelman, Michael R Hayden

**Affiliations:** 1 Centre for Molecular Medicine and Therapeutics, Child and Family Research Institute, Program in Neuroscience, University of British Columbia, Vancouver, V5Z 4H4, Canada; 2 Centre for Molecular Medicine and Therapeutics, Child and Family Research Institute, Department of Medical Genetics, University of British Columbia, Vancouver, V5Z 4H4, Canada; 3 The Mouse Imaging Centre, The Hospital for Sick Children, Toronto, M5T 3H7, Canada

**Keywords:** Huntington's Disease, neurodegeneration, caspase, magnetic resonance imaging

## Abstract

**Background:**

Huntington Disease (HD) is a neurodegenerative disorder in which caspase activation and cleavage of substrates, including the huntingtin protein, has been invoked as a pathological mechanism. Specific changes in caspase-2 (casp2) activity have been suggested to contribute to the pathogenesis of HD, however unique casp2 cleavage substrates have remained elusive. We thus utilized mice completely lacking casp2 (casp2-/-) to examine the role played by casp2 in the progression of HD. This 'substrate agnostic' approach allows us to query the effect of casp2 on HD progression without pre-defining proteolytic substrates of interest.

**Results:**

YAC128 HD model mice lacking casp2 show protection from well-validated motor and cognitive features of HD, including performance on rotarod, swimming T-maze, pre-pulse inhibition, spontaneous alternation and locomotor tasks. However, the specific pathological features of the YAC128 mice including striatal volume loss and testicular degeneration are unaltered in mice lacking casp2. The application of high-resolution magnetic resonance imaging (MRI) techniques validates specific neuropathology in the YAC128 mice that is not altered by ablation of casp2.

**Conclusions:**

The rescue of behavioral phenotypes in the absence of pathological improvement suggests that different pathways may be operative in the dysfunction of neural circuitry in HD leading to behavioral changes compared to the processes leading to cell death and volume loss. Inhibition of caspase-2 activity may be associated with symptomatic improvement in HD.

## Background

Huntington disease (HD) is a neurodegenerative disorder characterized by progressive motor, cognitive and psychiatric deficits [1] caused by an expanded poly-glutamine tract in the huntingtin (HTT) protein [2]. Neuropathologically, HD is characterized by early loss of medium spiny neurons (MSNs) in the striatum, accompanied by gliosis and eventual progressive neuronal loss throughout the brain [3].

Caspases are a family of proteases initially described to play critical roles in apoptotic cell death [4], whose non-apoptotic cellular functions are increasingly realized [5]. HTT has been shown to be a substrate *in vitro *for caspases-1,2,3 and -6 [6-9]. A number of additional proteins whose mutation causes neurodegeneration are also caspase substrates, including the amyloid precursor protein [10-12], tau [13], atrophin-1 [9], ataxin-3 [9], ataxin-7 [14] and the androgen receptor [9].

The commonality of caspase cleavage of neurodegenerative disease proteins could reflect their degradative clearance during cell death. Alternatively, these cleavage events could mediate apoptotic signaling, as is the case for bid [15], XIAP [16] and the caspases themselves [17]. Supporting the latter hypothesis, mutation of obligate aspartate residues in the caspase recognition regions of HTT has established that cleavage at amino acid 586, a caspase-6 site *in vitro*, is crucial for the development of HD symptoms in a mouse model [18-20]. Analogous rescue is observed in mutant APP-transgenic mice resistant to caspase cleavage at aspartate-664 [21,22]. Further, prevention of caspase processing of both atrophin-1 [23] and ataxin-7 [14] reduces toxicity *in vitro*. These experiments suggest that caspase-mediated cleavage of neurodegenerative disease proteins plays a role in the development of these conditions.

Caspase-2 (casp2) has been implicated in both Alzheimer's and Huntington disease. Dominant-negative casp2 constructs rescue mutant HTT-induced toxicity in rodent neurons, and total casp2 levels are increased in vulnerable neurons in human patient post-mortem tissue [6]. Furthermore, antisense to casp2 protects multiple neuronal cell lines from Aß1-42 induced toxicity [24-26]. Despite these disease associations, being the second mammalian caspase cloned [27,28] and its high degree of evolutionary conservation [29] the role of casp2 *in vivo *has remained unresolved.

While HTT itself is cleaved by casp2 at aspartate-552 *in vitro *[6], this event is not crucial for development of HD because mutating this site does not confer protection from HD symptoms in a mouse model [18], and constitutive cleavage at this site is observed in control, as well as diseased, brains [7]. However, experiments in rodent neurons with dominant-negative casp2 constructs support the idea that activity of casp2 may contribute to mutant-HTT induced toxicity [6]. While casp2 cleavage of HTT is thus unlikely to cause pathology in HD, other activities of casp2 may contribute to signs and symptoms of HD.

Very few casp2 cleavage substrates have been reported: casp2 [30], golgin-160 [31], αII-spectrin [32], protein kinase C (delta) [33], and Bid [34]. Of these events, only golgin-160 cleavage is uniquely catalyzed by casp2 [31]. Furthermore, the reagents commonly used as inhibitors and markers of casp2 activity based on the binding between VDVAD pseudo-substrate and active casp2 enzyme have been conclusively shown to be nonspecific [35], limiting their use in complex samples.

To examine the role of casp2 in HD we thus undertook a 'substrate agnostic' approach. Rather than focusing on particular potential cleavage events, we studied the development of HD symptoms in the well-validated YAC128 murine model of HD [36-38] when bred to casp2 -/- mice [39] to determine whether developmental and complete lack of casp2 may in some way modify the phenotype of HD, without relying on proxy makers for caspase activation.

## Results

### Casp2 mRNA and HTT protein levels in the brain

Casp2 has been described as transcriptionally upregulated in the striatum of the YAC72 mouse model of HD [6] and total casp2 immunoreactivity is increased in the brain of HD patients with significant striatal pathology [6]. To more directly examine caspase-2 transcription, we examined publically available microarray data [40] from early-stage (Vonsattel grade 0-2) human HD striatal tissue. This reveals no change in casp2 mRNA levels in the striatum of HD patients (table 1). Levels of transcripts reduced in HD, such as HMG-CoA reductase [41], are reduced in HD patients in this population, supporting the robustness of the dataset (table 1).

**Table 1:** Casp2 expression in the striatum of YAC128 mice and human HD patients

Species	Age (M)	Disease Stage*	Gene	Expression, HD/Control	SEM	t-value	p-value
Mouse	3		Casp2	.704	.063	1.78	> 0.05
Mouse	6		Casp2	1.15	.11	0.56	> 0.05
Mouse	9		Casp2	1.20	.12	0.71	> 0.05
Mouse	12		Casp2	1.01	.12	0.08	> 0.05
Human**		0-2	Casp2	0.996		-0.14	> 0.05
Human**		0-2	HMGCR	0.739		-5.04	< 0.001

Human striatal casp2 mRNA levels were extracted from publically available sources of microarray data (Affymetrix HG-U133A/B probe set 208050_s_at, HDBase.org-originally described in [66]). Mouse striatal casp2 levels were determined using QRT-PCR at 3, 6, 9 and 12 months of age. Casp2 is not upregulated in the striatum of aging YAC128 mice or human HD patients.

* Vonsattel neuropathological score at time of death [3]

** Human data extracted from [40]

Consistent with this human data, quantitative real-time PCR (QRT-PCR) of striatal casp2 mRNA demonstrates that wild type (WT) and YAC128 mice have equivalent levels throughout their life spans (table 1, two-way ANOVA, Genotype: F(1,27) = 0.073, p = 0.79; Age: F(3,27) = 2.48, p = 0.082; Interaction: F(3,27) = 2.71, p = 0.065). Thus, in both a rigorously characterized animal model of HD, as well as patient material, we see no evidence for transcriptional upregulation of casp2 in affected tissues at a time when both the mice and humans are displaying symptoms of HD.

We then considered whether levels of full-length mutant HTT could be altered by expression of casp2-/-. Using an antibody that recognizes long glutamine repeats (1C2, Millipore MAB1574), we performed western blots with cortices of 12-month old YAC128 and casp2-/-;YAC128 mice. There was no effect of genotype on full-length mutant huntingtin levels (data not shown, t(4) = 0.12, p = 0.91). We have previously demonstrated that the aa586 caspase-6 fragment of HTT is uniquely linked to toxicity in the YAC128 model of HD [18-20]. We determined the levels of the aa586 caspase-6 fragment in the cortex of 12 month old YAC128 and casp2-/-;YAC128 mice, using the same western blots. These blots reveal no effect of genotype on aa586 fragment levels (data not shown, t(4) = 0.36, p = 0.74).

### Casp2 -/- mice are protected from motor and cognitive symptoms of HD

The YAC128 murine model of HD robustly recapitulates many of the signs and symptoms of HD [37,38]. In order to examine the effect of casp2 on HD onset and progression, we bred heterozygous YAC128 [38] mice to a casp2 -/- [39] and WT background.

**Figure 1:**
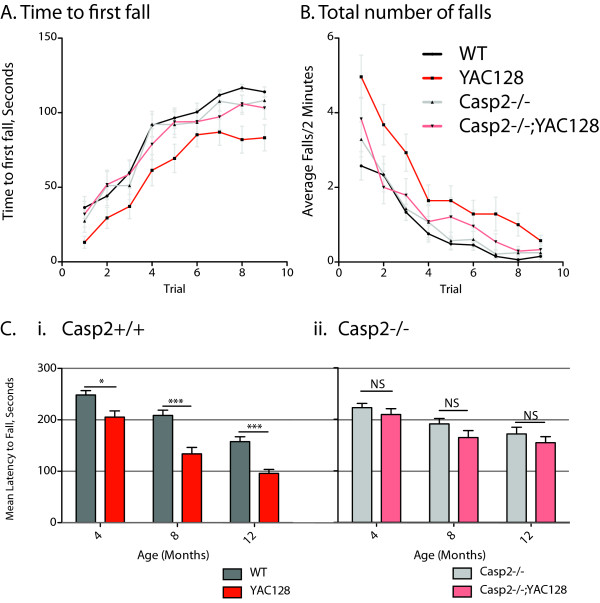
**Casp2-/- mice are protected from rotarod learning and accelerating rotorod deficits in the YAC128 mice**. A-B) Naïve mice were trained at 4 months of age on a fixed speed (18 RPM) rotorod; 9 trials of 2 minutes each were conducted over three days. The time to first fall and the number of falls during each trial was recorded. Casp2-/- mice are unaffected on this task, compared to WT littermates as measured by time to first fall, while YAC128 mice show significant impairment that is rescued in casp2-/-;YAC128 mice (linear mixed effects model YAC128 F(1,109) = 7.74, p = 0.0064; casp2 F(1,109) = 1.43, p = 0.23; Interaction F(1,109) = 5.36, p = 0.023) or number of total falls (linear mixed effects model YAC128 F(1,109) = 9.45, p = 0.0026; casp2 F(1,109) = 1.09, p = 0.30; Interaction F(1,109) = 3.97, p = 0.049). Data represent mean +/- SEM. N = 33 WT mice, 28 YAC128 mice, 28 casp2-/- mice and 24 casp2-/-;YAC128. C) Mice were tested on an accelerating rotorod (5-40 RPM) at 4, 8 and 12 months. Each mouse performed 3 5-minute trials and the mean of three trials recorded. YAC128 mice perform worse than WT littermates on this task (i-two-way ANOVA, YAC128 F(1,51) = 23.07, p < 0.0001, age F(2,51) = 77.92, p < 0.0001; interaction F(2,51) = 1.61, p = 0.20), while casp2-/-;YAC128 mice do not perform significantly worse than casp2-/- littermates (ii-two-way ANOVA, YAC128 F(1,45) = 2.39, p = 0.13, age F(2,45) = 18.0, p < 0.0001; interaction F(2,45) = 0.11, p = 0.89). Data represent mean +/- SEM. N = 33 WT mice, 28 YAC128 mice, 28 casp2-/- mice and 24 casp2-/-;YAC128.

From two months of age YAC128 mice demonstrate performance deficits when first exposed to a fixed-speed (18 RPM) 2-minute rotarod running task [37]. In this cohort, at 4 months of age, no effect of casp2 ablation is observed in WT mice on the time to first fall during 2-minute training sessions Figure 1A, gray and black lines). As described [37], YAC128 mice perform significantly worse than WT mice, an effect which is rescued in casp2-/-;YAC128 mice (Figure 1A, red and pink lines, linear mixed effects model YAC128 F(1,109) = 7.74, p = 0.0064; casp2 F(1,109) = 1.43, p = 0.23; Interaction F(1,109) = 5.36, p = 0.023). The mean time to first fall across all 9 trials is 68% of WT levels in the YAC128 mice, but 94% of WT levels in the casp2-/-;YAC128 mice, showing that casp2-/-;YAC128 mice are performing at essentially WT levels on this motor task. Examination of the total number of falls during the training session validates the specific impairment of the YAC128 mice, which is again ameliorated in the casp2-/-;YAC128 mice (Figure 1B, linear mixed effects model YAC128 F(1,109) = 9.45, p = 0.0026; casp2 F(1,109) = 1.09, p = 0.30; Interaction F(1,109) = 3.97, p = 0.049).

After training, mice were tested on a 5-minute accelerating rotarod (5-40 RPM) task at 4, 8 and 12 months of age. As previously shown, YAC128 mice perform significantly worse than littermates on this task (Figure 1C i, two-way ANOVA, YAC128 F(1,102) = 23.07, p < 0.0001, Age F(2,102) = 77.92, p < 0.0001, Interaction F(2,102) = 1.61, p = 0.24). In mice lacking casp2, this effect is ameliorated (Figure 1C ii, two-way ANOVA, Genotype F(1,90) = 2.4, p = 0.13, Age F(2,90) = 18.0, p < 0.0001, Interaction F(2,90) = 0.11, p = 0.89). At 12 months of age, YAC128 latency to fall is reduced by 39% compared to WT mice (95.8 sec. vs. 157.6 sec, respectively). Casp2-/-;YAC128 mice remain on the rotarod for an average of 154.6 seconds, 98% of WT levels, suggesting complete rescue of this phenotype.

**Figure 2:**
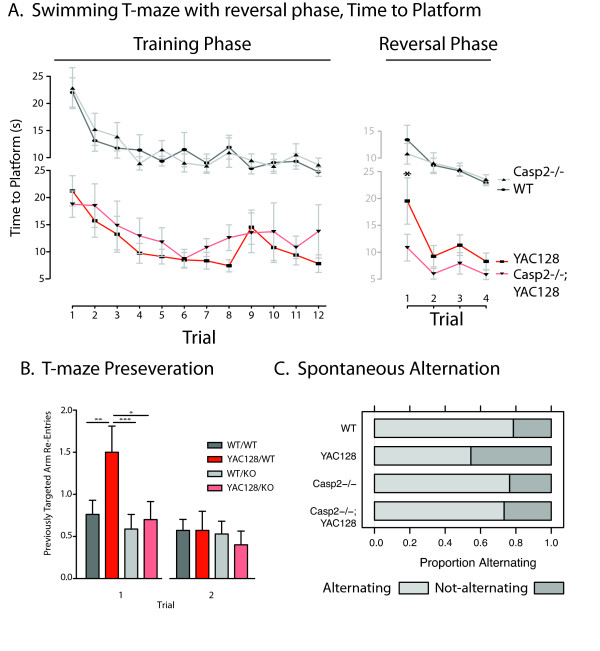
**Casp2-/- mice are protected from cognitive symptoms of HD in the YAC128 mouse**. A) Mice were trained in a swimming T-maze to find a submerged platform in one arm of the maze. Acquisition time of the platform location does not differ between genotypes during the first 12 trials (two-way repeated measures ANOVA genotype: F(3,638) = 0.56, p = 0.64). Before trial 1 of the reversal phase on day 5, the platform was switched to the opposite arm of the T-maze. Time to the platform in its new location differed by genotype across the 4 trials (two-way repeated measures ANOVA, genotype: F(3,147) = 2.90, p = 0.043). YAC128 mice take significantly longer to reach the platform on the first trial than Casp2-/-;YAC128 mice (19.5 seconds vs. 10.86 seconds, Bonferroni t = 3.205, p < 0.01). Data represent mean +/- SEM. N = 33 WT mice, 28 YAC128 mice, 28 casp2-/- mice and 24 Casp2-/-;YAC128 mice. B) Increased time to platform in the YAC128 mice was primarily due to increased perseveration during the first trial. YAC128 mice re-entered the previously correct arm more frequently than Casp2-/-;YAC128 mice (two-way repeated measures ANOVA Trial F(3,174) = 10.19, p < 0.0001; YAC128 F(3,174) = 2.04, p = 0.12; Interaction F(9,174) = 1.42, p = 0.18; Bonferroni post-hoc test significance indicated). N = 21 WT mice, 28 YAC128 mice, 17 casp2-/- mice and 24 Casp2-/-;YAC128 mice. C) YAC128;casp2-/- are rescued from deficits in a T-maze spontaneous alternation task. 7-month old Mice were exposed to a T-maze with a divider forcing them to choose one arm, which they were restrained to for 1 minute. After this familiarization trial, the mice re-entered the T-maze and their arm choice recorded. WT mice prefer the novel arm of the maze 79% of the time, while YAC128 mice only choose the novel arm 53% of the time. Casp2-/- and YAC128;casp2-/- choose the novel arm 77% and 73% of the time, similar to WT mice. N = 15 WT mice, 21 YAC128 mice, 17 casp2-/- mice and 15 Casp2-/-;YAC128 mice.

In addition to motor impairment, YAC128 mice demonstrate clear cognitive and psychiatric deficits analogous to those seen in human HD patients [37,42,43]. To probe perseverative behaviors, we used a previously validated swimming T-maze task [37]. At 12 months of age there is no difference in swimming speed between any of the genotypes in the current study, and all four genotypes of mice learned to reach a submerged platform equally well during 12 training runs over 4 days (Figure 2, left side, two-way repeated measures ANOVA, genotype: F(3,58) = 0.56, p = 0.64). On day 5, the platform was switched to the opposite arm of the swimming t-maze and YAC128 mice require significantly longer to reach the new platform location, while casp2-/-;YAC128 mice perform similar to WT mice (Figure 2A, right side, two-way repeated measures ANOVA, genotype: F(3,58) = 2.90, p = 0.043, Bonferroni post-test trial 1, Casp2-/-;YAC128 vs. YAC128, t = 2.78, p < 0.05). This is primarily due to increased perseveration during the first reversal trial-the YAC128 mice re-enter the previously correct arm of the maze more frequently than YAC128 mice lacking casp2 (Figure 2B, repeated measures two-way ANOVA Bonferroni post-hoc tests indicated). These data suggest that YAC128 mice lacking casp2 are protected from loss of cognitive flexibility-a cardinal early feature of psychiatric disturbances in HD [44,45].

To extend our understanding of the cognitive defects underlying this deficiency, we examined the spontaneous alternation of mice in a T-maze [46]. In this spatial memory task mice are released at the base of the T-maze and allowed to choose an arm to explore. When re-exposed to the maze, normal mice recall the previously visited arm of the T-maze and prefer to investigate the novel arm 79% of the time. YAC128 mice perform near chance in this task, visiting the new arm of the maze only 53% of the time. Both casp2-/- and casp2-/-;YAC128 mice perform similarly to WT mice choosing the new arm 77% and 73% of the time respectively (Figure 2C).

**Figure 3:**
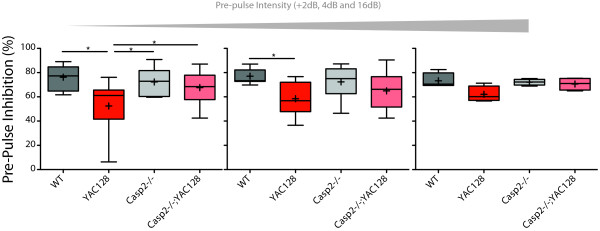
**Casp2-/-;YAC128 mice are protected from pre-pulse inhibition deficits**. Mice were tested for pre-pulse inhibition at 12 months of age. YAC128 mice show reduced PPI, compared to littermates, while casp2-/-;YAC128 mice do not (one-way ANOVA 2dB Genotype F(3,39) = 4.49, p = 0.0089; 4dB Genotype F(3,38) = 3.07, p = 0.04; 16dB Genotype F(3,37) = 2.25, p = 0.10; Newman-Keuls post-hoc test p-values indicated). Mean="+", horizontal bars = quartiles. N = 7 WT mice, 12 YAC128 mice, 9 casp2-/- mice and 12 Casp2-/-;YAC128 mice. Post-hoc Bonferroni genotype comparisons "*" indicates p < 0.05.

HD patients show reduced pre-pulse inhibition (PPI) [47], as do YAC128 mice [37]. At 12 months of age startle amplitude in response to a 120dB noise is equivalent in all examined mice (one-way ANOVA, genotype: F(3,37) = 0.36, p = 0.78). In a PPI paradigm with a background noise + 2dB, 4dB or 16dB warning tone the YAC128 mice have reduced PPI compared to WT mice (Figure 3B, 2dB Genotype F(3,39) = 4.49, p = 0.0089; 4dB Genotype F(3,38) = 3.07, p = 0.04; 16dB Genotype F(3,37) = 2.25, p = 0.10; Newman-Keuls post-hoc test p-values indicated). Lower intensity pre-pulses led to more obvious deficits in the YAC128 mice, in agreement with previously published results [37]. This effect was ameliorated in casp2-/-;YAC128 mice, who had normalized PPI compared to YAC128 mice, suggesting that sensorimotor gating defects are corrected in YAC128 mice lacking casp2.

**Figure 4:**
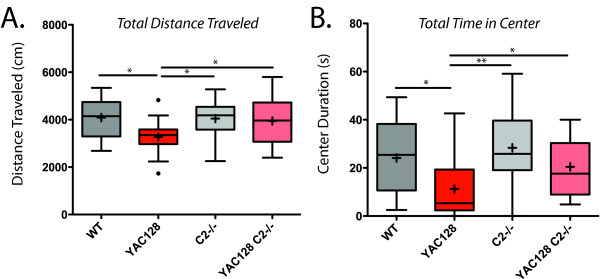
**Casp2-/- mice are protected from locomotor symptoms of HD in the YAC128 mouse**. At 7 months of age, YAC128 mice display decreased locomotor activity and increased anxiety, as measured by time spent in the center of the arena, during exploration of an open field as compared to WT mice. Casp2-/- and casp2-/-;YAC128 mice perform similarly to WT mice. Mean="+", horizontal bars = quartiles. N = 15 WT mice, 21 YAC128 mice, 17 casp2-/- mice and 15 Casp2-/-;YAC128 mice. Post-hoc Newman-Keuls genotype comparisons "*" indicates p < 0.05.

YAC128 mice are hypoactive during exploration of an open field after about 6 months of age [38]. This locomotor phenotype is rescued in the casp2-/-;YAC128 mice at 7 months of age (Figure 4A, one way ANOVA F (3,67) = 4.30, p = 0.0079, Newman-Keuls post-hoc tests indicated). In addition to their hypoactive phenotype, the YAC128 mice show anxiety by spending less time in the center of the open field arena, and entering the center zone less frequently [48]. This phenotype is also ameliorated in the casp2-/-;YAC128 mice (Figure 4B, right, one way ANOVA F (3,67) = 5.73, p = 0.0015, Newman-Keuls post-hoc tests indicated).

### Casp2-/- mice are not protected from pathological features of HD

**Figure 5:**
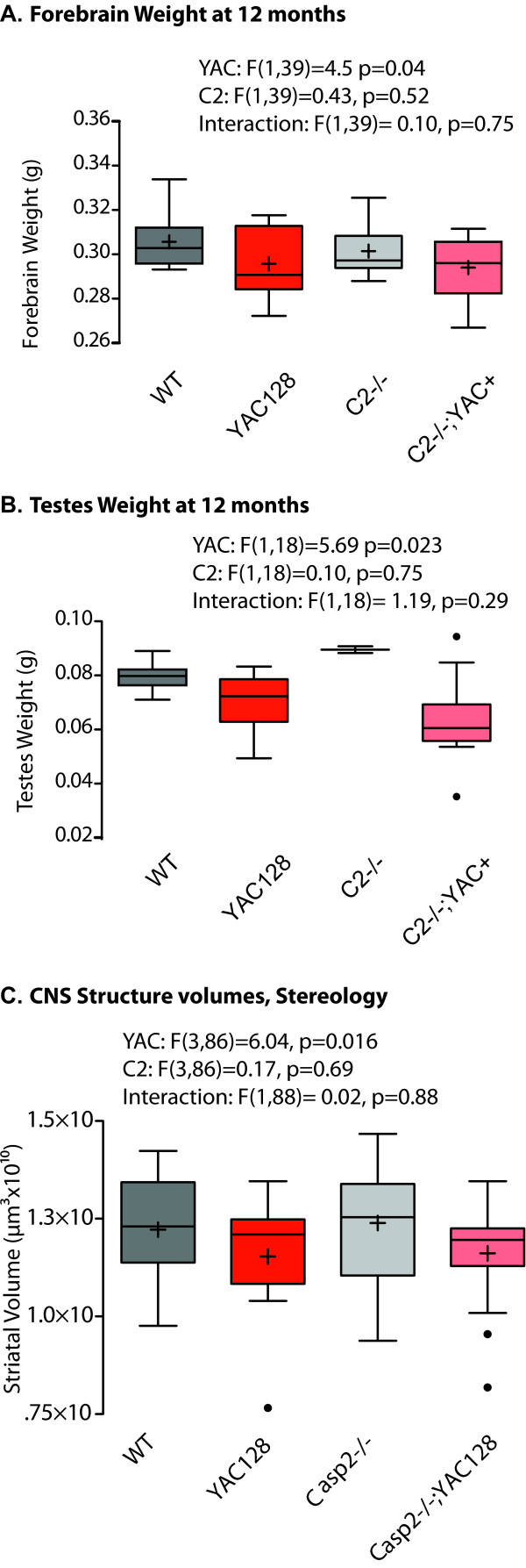
**Casp2-/- mice are not protected from pathology**. A-B) Fixed forebrain and testes weight in 12-month-old mice. YAC128 mice show forebrain atrophy that is not rescued by the absence of casp2 (summary statistics for two-way ANOVA indicated). Mean="+", horizontal bars = quartiles. N = 9 WT mice, 11 YAC128 mice, 9 casp2-/- mice, 14 Casp2-/-;YAC128 mice. C) Striatal volume, as determined by stereology, is reduced in YAC128 mice and not affected by caspase-2 expression. Mean="+", horizontal bars = quartiles, isolated dots = outliers. Factorial ANOVA F/p-values indicated. N = 19 WT mice, 25 YAC128 mice, 21 casp2-/- mice, 22 Casp2-/-;YAC128 mice.

Brain weight is the simplest measure of neurodegeneration, and is decreased in the YAC128 mice [38]. In the present cohort, forebrain weight is reduced in the YAC128 mice, an effect which is not ameliorated by the absence of casp2 (Figure 5A, linear mixed effects model, YAC128 F(1,39) = 4.5, p = 0.040; casp2 F(1,39) = 0.432, p = 0.52). Male YAC128 mice and human HD patients also have specific testicular degeneration [49], a phenotype which is also not affected by the absence of casp2 (Figure 5B, linear mixed effects model, YAC128: F(1,18) = 5.70, p = 0.028; Casp2: F(1,18) = 0.10, p = 0.75). Like HD patients, YAC128 mice have progressive specific striatal volume decreases [38,50]. Using stereological techniques we observe reduced striatal volume in the YAC128 mice that is not ameliorated in the casp2-/-;YAC128 mice (Figure 5C, two-way ANOVA YAC128: F(3,86) = 6.04, p = 0.016; casp2 F(3,86) = 0.17, p = 0.69; Interaction F(1,88) = 0.02, p = 0.88).

To gain more detailed information on YAC128 neuropathology, we have developed and described magnetic resonance imaging (MRI) techniques to generate volumetric data for a number of CNS structures [50-53]. These techniques allow the complete three-dimensional delineation of structures, avoiding artificial truncation due to tissue processing limitations. Measurements are conducted on brains *in situ *in the skull, minimizing processing and handling artifacts. Also, using atlas based MRI techniques it is possible to simultaneously determine volumes for a large number of structures, rather than limiting analyses to pre-determined regions of interest. Direct comparison of MRI and stereological approaches to measuring striatal volume in the YAC128 mice has shown that while the volume loss detected by each technique is the same, MRI is more accurate and therefore powerful [50,54].

**Figure 6:**
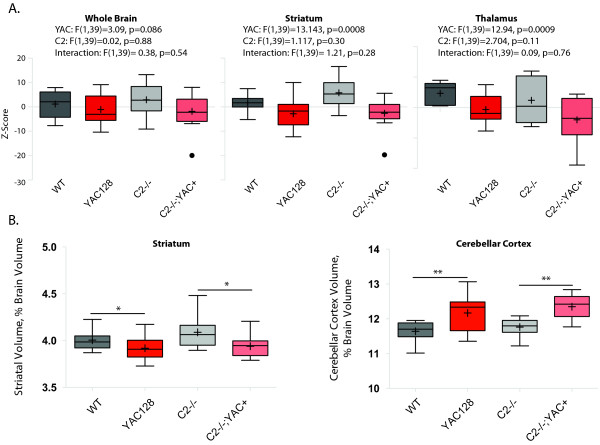
**Casp2-/- mice are not protected from brain pathology assayed with MRI**. A) Volumes for brain structures were determined by automated segmentation of MRI images and expressed as Z-scores. YAC128 mice have smaller brains, with specific shrinkage in the basal ganglia and other sub-cortical gray matter structures. Casp2-/- mice are not protected from any of these losses (summary statistics for two-way ANOVA indicated). B) To control for overall brain atrophy, structure volumes were expressed as a percentage of brain volume. Relative striatal volume is decreased in the YAC128 mice, and not rescued in the casp2-/-;YAC128 mice (Post-hoc Bonferroni genotype comparisons "*" indicates p < 0.05). The cerebellum is relatively preserved in the YAC128 mice, due to selective forebrain degeneration. This phenotype is not ameliorated in the casp2-/-;YAC128 mice (Post-hoc Bonferroni genotype comparisons "*" indicates p < 0.05). Mean="+", horizontal bars = quartiles, isolated dots = outliers. N = 9 WT mice, 11 YAC128 mice, 9 casp2-/- mice, 14 Casp2-/-;YAC128 mice.

MRI data was acquired post-mortem, at twelve months of age-the same mice were used for both behavioral and MRI experiments. Total brain volume, as determined by MRI, was reduced in the YAC128 mice and not rescued in the casp2-/- mice, though reductions observed did not reach significance (Figure 6A, left, two-way ANOVA YAC128: F(1,39) = 3.10, p = 0.086; casp2: F(1,39) = 0.02, p = 0.88; Interaction F(1,39) = 0.38, p = 0.54). MRI-detected striatal volume is specifically reduced in the YAC128 mice, and not affected by casp2 expression (Figure 6A, two-way ANOVA YAC128: F(1,39) = 13.14, p = 0.0008, casp2: F(1,39) = 0.02, p = 0.85; Interaction: F(1,39) = 0.38, p = 0.54). Other brain structures, such as the thalamus, show atrophy in the YAC128 mice [54,50], and human patients [55]. This atrophy was validated in the current cohort and was not affected by casp2 expression (Figure 6A, two-way ANOVA, YAC128: F(1,39) = 11.41, p = 0.0017; casp2: F(1,39) = 2.704, p = 0.11; Interaction: F(1,39) = 0.09, p = 0.76).

MRI-detected volumes are valuable measures of tissue atrophy because they can be normalized to total brain volume. These "brain normalized" values highlight specific regions of atrophy and preservation, by correcting for changes in brain volume. We have shown that regions of both atrophy (the striatum) and preservation (the cerebellum) can be observed with these measures in the YAC128 mice [50]. In agreement with the stereological data, striatal volume, when considered as a fraction of total brain volume, is reduced in the YAC128 mice, and not rescued in the casp2-/-;YAC128 mice (Figure 6B, Bonferroni post-hoc tests indicated). Analogously, the cerebellar cortex is increased in relative volume, due to its preservation in the face of HD-induced atrophy. This cerebellar preservation is not altered in the casp2-/-;YAC128 mice (Figure 6B, Bonferroni post-hoc tests indicated). These results validate the specific pathological tissue loss in the YA128 mice, the use of MRI to ascertain these losses, and clearly demonstrate that the absence of casp2 is not associated with amelioration of this loss.

## Discussion

The primary finding of the current study is that casp2-/- mice are protected from behavioral and cognitive features of HD in the YAC128 model. It is remarkable that every behavioral and cognitive feature of HD examined is completely rescued in the absence of caspase-2. However, pathological phenotypes of HD including specific striatal volume loss and testicular degeneration are not rescued in the casp-2-/- mice.

### Caspase-2 transcription

This work expands our understanding of the activation of casp2 in HD. An earlier report suggested that transcriptional up-regulation, secondary to BDNF loss, could explain increases in casp2 levels in HD [6]. The data presented here do not support this hypothesis-human microarray data indicates that striatal casp2 levels are normal in early stage HD patients. Additionally, striatal casp2 mRNA levels in YAC128 HD mice are equivalent to WT levels during the development of HD-like behavioral changes. Thus, increased casp2 transcription is unlikely to contribute to the development of HD.

### Caspase-2 -/- mice are protected from motor and cognitive features of HD

Casp2-/- mice are protected from motor and cognitive deficits seen in the YAC128 model of HD. Consistent with previous reports, we show that the YAC128 mice have deficits during the learning phase of the rotarod task at 4 months of age [37]. This learning deficit is completely reversed in the YAC128 mice lacking casp2, who are also protected from progressive motor impairment on the previously learned task. This demonstrates that casp2-/-;YAC128 mice are protected from both cognitive and motor aspects of this task.

Data from the pre-pulse inhibition, swimming T-maze and spontaneous alternation tasks presented here are consistent with the cognitive flexibility deficits previously observed in the YAC128 mice [37]. All these tasks were performed significantly better by casp2-/-;YAC128 mice, relative to YAC128 mice. This suggests that YAC128 mice lacking casp2 are better able to modify their behavior in response to environmental demands than normal YAC128 mice. Specific deficits in behavioral flexibility are a key feature of the cognitive defects observed in human HD patients [56,57], and improvement of these symptoms in casp2-/-;YAC128 mice supports the idea that casp2 inhibition may have impact on neural function, leading to symptomatic benefit.

### Casp2-/- mice are not protected from pathological features of HD

Despite their protection from behavioral and cognitive symptoms, the volume loss observed in the YAC128 mice in structures such as the striatum and thalamus is not ameliorated by the absence of casp2. In the periphery, pathological atrophy of the testes is also not ameliorated in casp2-/-;YAC128 mice. The improved performance of the casp2-/-;YAC128 mice on behavioral tasks in the face of this pathological tissue loss suggests that neuronal circuits mediating disease relevant behavior are capable of augmentation leading to symptomatic benefit. These experiments demonstrate that loss of volume is not sufficient to cause all motor and cognitive features of HD in mice.

Previous interventions in the YAC128 mice have shown dissociation between neuropathological and behavioral endpoints. Symptomatic treatment with cystamine results in neuroprotection without affecting behavioral symptoms of HD [58]. Conversely, symptomatic treatment with ethyl-eicosapentaenoic acid provides relief from motor symptoms of HD, without altering neuropathological features [59]. These results, in conjunction with the current study, demonstrate that some aspects of pathological development are dissociable from tissue atrophy in HD. This dissociability of pathological and behavioral symptoms highlights the potential need for different approaches to treatment at different stages of illness, and also for different signs and symptoms. The value of symptomatic therapy is clear; tetrabenazine, the only FDA-approved drug for HD, is used as an anti-chorea agent with no claim of disease modification [60].

## Conclusions

Casp2-/- mice are protected from a number of behavioral features of HD in the YAC128 mouse model. This protection is seen in a number of paradigms, including motor learning, motor coordination, cognitive flexibility, spatial learning, sensorimotor gating and locomotor behavior. Casp2-/- mice are not protected from pathological signs of HD including testicular atrophy and regionally specific brain atrophy as assayed by stereology and magnetic resonance imaging techniques. These findings lend weight to the concept that symptomatic improvement is possible in HD even in the face of unaltered pathology.

A secondary goal of the current study was to objectively evaluate the usefulness of casp2 as a drug target in HD. There are several caveats to careful interpretation of these data. First, in these mice casp2 expression is absent throughout development, rather than being postnatally suppressed, as with a drug. Also, we have completely ablated, rather than reduced, the activities of casp2. Therefore it is difficult to extrapolate these findings with a genetic model to the effect of a drug, but these beneficial effects clearly warrant additional investigation. The single mutation underlying HD has a large array of effects on cells, and if the mutant HTT protein cannot be directly targeted for therapy, it may be necessary to target multiple aberrant processes to provide effective therapy for HD. Based on findings here, casp2 inhibition coupled with neuroprotective therapy may be an effective strategy to combat behavioral, cognitive and neuropathological features of HD.

## Methods

### Mice and breeding

Caspase-2-/- mice [39] were obtained on the C57Bl/6 strain, and backcrossed for at least 7 generations to the FVB/NJ strain before being used for experiments or bred to the YAC128 mouse. YAC128 (line 53) mice [38] were maintained on the FVB/NJ strain. Caspase-2+/-;YAC128+/- breeders were intercrossed and pups of appropriate genotypes selected from the resultant progeny, to ensure subject mice were littermates. The resulting mice (wild type, casp2-/-, YAC128 and casp2-/-;YAC128) were genotyped for the *rd *allele which causes progressive retinal degeneration and blindness in the FVBN/J strain-all mice carried the FVBN/J *rd *mutant allele. Mice were genotyped for the YAC128 transgene and housed as previously described [38], and all animal experiments were conducted in accordance with protocols approved by the University of British Columbia Committee on Animal Care.

### Quantitative Real-time PCR (QRT-PCR)

Total RNA was extracted from dissected striata, frozen and stored at -80C using the RNeasy mini kit (Qiagen, 74104). cDNA was prepared using 250 ng total RNA and the superscript-III first-strand synthesis kit with oligo-dT priming (Invitrogen, 11752-050). Primers used included mouse casp2 forward: 5'-GAATGAACCTTATCGGGCATAACT-3' and reverse: 5'-GATGACGGGTGATAGTGTGAGACA-3'. Mouse actin forward: 5'-ACGGCCAGGTCATCACTATTG-3' and reverse: 5'-CAAGAAGGAAGGCTGGAAAAGA-3'. QRTPCR was conducted using SYBR Green PCR master mix (Applied Biosystems, 4309155) in the ABI7500 instrument (Applied Biosystems) using the absolute quantification standard curve method.

### Behavioral assays

Mice were single housed in microisolator cages with a 12 h light/dark cycle. Mice were randomly coded and the experimenter was blind to genotype. Motor coordination and learning were examined using an accelerating rotarod (UGO Basile, Comerio, Italy). For training, naïve 4-month-old mice were given three trials of 2 minutes on a fixed speed (18 RPM) task per day for three days (9 trials total). The inter-trial interval was 2 hours. Mice falling from the rod were returned, to a maximum of 10 falls/trial. The time to first fall and total number of falls per trial were recorded. For longitudinal accelerating rotarod assessment 4-, 8- or 12-month-old mice were tested on a rod accelerating from 5 to 40 RPM over 300 seconds. Latency to fall from the rod was recorded. 3 trials in 1 day were averaged to give mean performance for each mouse at each age.

Acoustic startle and prepulse inhibition (PPI) were measured in SR-LAB chambers (San Diego Instruments, San Diego, USA). Before use, the chambers were calibrated using a vibrating standardization unit at 700 V (San Diego Instruments, San Diego, USA). After a 5-minute acclimatization period mice were exposed to 100 50 ms startle stimuli with intensities ranging from background level (70 dB) to 120 dB. Startle stimuli were presented in pseudorandomized order in 10 blocks of 10 trials, with a pseudorandomized 8-32 second inter-trial interval.

For the PPI task, the "pulse" was a 40 ms 120 dB stimulus. Eight blocks of trials were conducted-the first and last of which were a series of 6 pulse only trials. The first block was used to determine the average startle intensity. The subsequent 6 blocks consisted of 6 trials: 70 dB (background) alone ("no-pulse"), 120 dB for 40 ms alone ("pulse") and 4 pre-pulse trials with pre-pulse intensities of 72, 74, 78 and 86 dB (20 ms duration, pre-pulse interval of 100 ms). PPI was calculated as: [(First 120 dB block response)-(PPI block response)]/(First 120 dB block response). PPI stimuli were presented in pseudorandomized order with a 8-32 second inter-trial interval.

For the swimming T-maze + reversal task mice were tested in a white acrylic maze with arm dimensions 38 × 14 cm [37]. The maze was filled with water and a platform (10 × 14 cm) submerged below the water surface in one arm of the maze. Mice were released at the base of the stem of the T and learned to swim to the submerged platform-the time to platform, total number of arm entries and arm re-entries were recorded. For training, mice received 4 trials per day for 3 days (12 total trials) with a 45-minute inter-trial interval. On the 5th day, the platform was switched to the opposite arm of the maze and mice were required to change strategies to find the platform in its new location. Mice received 4 trials with a 45-minute inter-trial interval.

For the spontaneous alternation task mice were tested in the maze described above for the swimming T-maze task without water. A dividing wall was placed at the intersection of the T extending into the stem of the T to encourage entry into a single goal arm. Mice were placed at the base of the T and allowed to choose a goal arm. A barrier was then lowered to prevent exit from the goal arm during a 1 minute exploration interval during which the central divider was removed. Mice were then removed from the explored goal arm and immediately placed at the base of the T and once again allowed to choose a goal arm. Mice who entered the previously explored goal arm were given a score of 0 while mice who entered the novel arm were given a score of 1.

For the open field exploration task mice were tested in a gray acrylic open topped box with dimensions 50 × 50 × 20 cm under bright lighting. Mice were placed in the corner of the box and allowed to explore for 10 minutes. Trials were recorded using a ceiling mounted video camera and exploration tracks were analyzed using EthoVision XT 7.0 software (Noldus).

### Pathology, including MRI

Mice were terminally anesthetized by intraperitoneal injection of 2.5% avertin, and transcardially perfused with 30 mL phosphate buffered saline, followed by 30 mL 4% ice-cold paraformaldehyde in PBS. Testes were dissected out and weighed after fixation. Heads were removed and skin, lower jaws ears and cartligenous nose tip dissected away. Skulls were post-fixed in 4% paraformaldehyde at 4°C overnight. Skulls were soaked in PBS + 0.01% NaAzide for 5 days at room temperature with rotation. Skulls were then enhanced in PBS + 0.02% NaAzide with 2 mM Prohance (Bracco Diagnostics, DIN 02229056). A 7.0 Tesla MRI scanner (Varian Inc., Palo Alto, CA) with a 6 cm inner bore diameter insert gradient set was used for all MRI scans. Parameters used for anatomical MRI scans were optimized for high efficiency and gray/white matter contrast: T2-weighted, 3D fast spin echo, with a TR of 325 ms, and TEs of 10 ms per echo for 6 echoes, four averages, field-of-view of 14 × 14 × 25 mm^3 ^and matrix size of 432 × 432 × 780 giving an image with 0.032 mm isotropic voxels. Total imaging time for this MRI sequence is ~12 hours [61]. Volumes were then automatically segmented into 62 separate anatomical structures using automated image registration techniques [62-64]. For graphing, volumes were standardized and expressed as Z-scores. After imaging skulls were removed and brains weighed.

### Statistics

For data with one independent variable an unpaired t-test or one-way ANOVA model was fitted and tested (as appropriate), followed by Newman-Keuls or Bonferroni post-hoc tests. Data with two or more independent variables was analyzed by two-way ANOVA. To control for repeated measurements and other sources of correlated error data were analyzed using repeated measures two-way ANOVA or, when values were missing, by fitting a linear mixed effects (LME) model, followed by analysis of variance testing. In LME models, each mouse was assigned a random intercept. T-tests, one- and two-way ANOVA's were performed using Prism 4.0 software, and linear mixed effects models were done using the R language and environment including the "nlme" linear mixed effects model package [65].

## List of Abbreviations

ANOVA: analysis of variance; LME: linear mixed effects model; Casp2: caspase-2; HD: Huntington's Disease; Htt: Huntingtin (gene, PROTEIN); YAC128: Yeast artificial chromosome, 128 CAG repeats; PPI: Pre-pulse inhibition; MRI: Magnetic resonance imaging.

## Funding

This work was supported by the Michael Smith Foundation for Health Research [ST-SGS-00835(06-1)BM, 00495(06-1)BM], Canadian Institutes of Health Research [CGD-85375], Huntington Society of Canada [Landmark Graduate Award, 2005] and CHDI Inc. [TREAT-HD].

## Competing interests

The authors declare that they have no competing interests.

## Authors' contributions

JBC conceived and designed the study, performed behavioral, immunohistochemical and statistical analyses and drafted the manuscript. ALS performed behavioral experiments, statistical analyses and aided in drafting the manuscript. WNZ performed behavioral analyses. RKG, DEE participated in the conception and design of the study, and contributed to the draft of the manucript. YD performed molecular experiments confirming genotypes of mice. NB perfused the mice used in the study and managed the animal colony. JPL and RMH conducted MRI analyses and generated volumetric data from brain regions, using a novel suite of tools. MRH conceived and designed the study and drafted the manuscript. All authors have read and approved the final manuscript.
